# In vitro, in vivo, and in silico analysis of synbiotics as preventive interventions for lipid metabolism in ethanol-induced adipose tissue injury

**DOI:** 10.1186/s12944-023-01809-z

**Published:** 2023-04-13

**Authors:** Dhara Patel, Pooja Rathaur, Kirti Parwani, Farhin Patel, Dixa Sharma, Kaid Johar, Palash Mandal

**Affiliations:** 1 P D Patel Institute of Applied Sciences, Charotar University of Science and Technology, Changa-388421 Gujarat, India; 2 Department of Life Science, School of Sciences, Gujarat University, Ahmedabad, 380009 Gujarat India; 3 Department of Zoology, Biomedical Technology, and Human Genetics, School of Sciences, Gujarat University, Ahmedabad, 380009 Gujarat India

**Keywords:** Adipose tissue, Alcoholic liver disease, Garlic, *Lactobacillus*

## Abstract

The risk of alcoholic liver disease (ALD) is increased by excessive ethanol drinking. For the prevention of ALD, the effects of ethanol on the liver, adipose tissue, and gut are crucial. Interestingly, garlic and a few probiotic strains can protect against ethanol-induced hepatotoxicity. However, the relationship between adipose tissue inflammation, Kyolic aged garlic extract (AGE), and *Lactobacillus rhamnosus* MTCC1423 in developing ALD is unknown. Therefore, the present study explored the effect of synbiotics (a combination of prebiotics and probiotics) on adipose tissue to prevent ALD. To investigate the efficacy of synbiotics administration on adipose tissue in preventing ALD, in vitro (3T3-L1 cells, *N* = 3) groups: control, control + LPS (lipopolysaccharide), ethanol, ethanol + LPS, ethanol + synbiotics, ethanol + synbiotics + LPS; in vivo (Wistar male rats, *N* = 6) groups: control, ethanol, pairfed, ethanol + synbiotics and in silico experiments were conducted. *Lactobacillus* multiplies in accordance with the growth curve when exposed to AGE. Additionally, Oil red O staining and scanning electron microscopy (SEM) demonstrated that synbiotics therapy maintained the morphology of adipocytes in the alcoholic model. In support of the morphological changes, quantitative real-time PCR demonstrated overexpression of adiponectin and downregulation of leptin, resistin, PPARγ, CYP2E1, iNOS, IL-6, and TNF-α after administration of synbiotics compared to the ethanol group. In addition, MDA estimation by high-performance liquid chromatography (HPLC) indicated that the synbiotics treatment reduced oxidative stress in rat adipose tissue. Consequently, the *in-silico* analysis revealed that AGE inhibited the C-D-T networks as PPARγ acting as the main target protein. The current study demonstrates that using synbiotics improves adipose tissue metabolism in ALD.

**Graphical Abstract:**

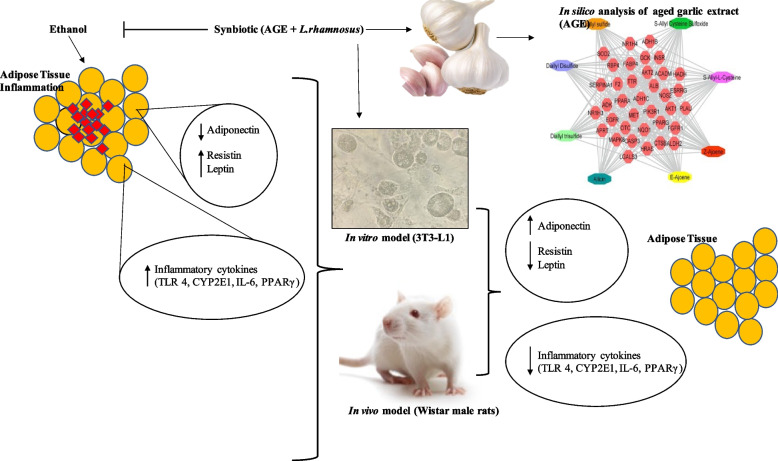

## Introduction

The hepatic injury spectrum in ALD ranges from fatty liver to steatosis to cirrhosis [1]. Hepatic steatosis is diagnosed when intrahepatic triacylglycerol accounts for a minimum of 5% of the liver's weight. Hepatic steatosis is caused mainly by ALD and nonalcoholic fatty liver disease (NAFLD). ALD accounts for 0.9% of deaths worldwide and 49% of cirrhosis mortality [2, 3]. In 2012, alcohol was responsible for 0.139 billion disability-adjusted life years (DALYs) and 5.9% of worldwide deaths [4]. Cirrhosis is caused by excessive drinking of alcohol. Ethanol metabolism is regulated by the enzymes cytochrome-P450-2E1 (CYP2E1), alcohol dehydrogenase, and aldehyde dehydrogenase. Alcohol metabolism through CYP2E1 leads to oxidative stress, endoplasmic reticulum (ER) stress, and an imbalance in adipokine secretions. According to reports, alcohol increases the expression of CYP2E1, leading to inflammation and oxidative stress, which all enhance cell toxicity and the development of ALD [5, 6]. Oxidative stress is caused by the activation of the CYP2E1-dependent ethanol pathway, leading to necrosis of hepatocytes due to a shift in the regulation of inflammatory genes such as tumor necrosis factor alpha(TNF-α), interleukin-6 (IL-6), toll like receptor-4 (TLR4), resistin, and leptin at the transcriptional level [7].

The liver, adipose tissue, and intestine are involved in the pathophysiology of ALD [8–13]. Sterol regulatory element binding protein (SREBP)-1c is crucial in developing hepatic lipogenesis and insulin resistance in mice chronically exposed to ethanol [14, 15]. The enzymes adenosine monophosphate-activated protein kinase (AMPK) and peroxisome proliferator-activated receptor (PPAR) are involved in the progression of hepatic steatosis [16, 17]. Recent findings indicate that body mass index and visceral fat deposition are essential in developing ALD [18–21]. Prolonged alcohol consumption is linked with decreased adiponectin levels, activated CYP2E1, lipolysis, oxidative stress, insulin resistance, and apoptosis in adipose tissue [22–25]. White adipose tissue (WAT) is substantially decreased due to hepatic steatosis in ALD by an upsurge in lipolysis that causes free fatty acids to flow into the blood [26]. According to a study, moderate amounts of fat and alcohol may induce hepatic steatosis [27].

The gut plays a crucial role in the pathophysiology of ALD. A decline in *Lactobacillus* species in the gut has been observed in alcoholic cirrhosis. Bacterial products such as lipopolysaccharide (LPS) have been reported in the blood of alcoholics [28, 29] because of intestinal mucosa-to-intrahepatic LPS translocation. This causes TLR4 activation in the liver and the development of ALD from steatosis to steatohepatitis [30–33]. Nondigestible carbohydrates benefit the host by altering the gut microbiota, and their activity is grouped into prebiotics [34]. Probiotics are defined as viable bacteria in sufficient quantities that have good health benefits on the host [35]. Probiotics such as *Lactobacillus* species adhere to intestinal epithelial cells, thereby inhibiting pathogens belonging to the *Enterobacteriaceae* family [36, 37]. Epithelial cells are nourished by short-chain fatty acids released by these bacteria during the fermentation phase of their metabolism [38]. The combination of prebiotics and probiotics is known as a synbiotics. Synbiotics are the future of probiotics, as they can modulate intestinal permeability, microflora, and the inflammatory response [39].

Only a few synthetic inhibitors, such as rosiglitazone (PPARγ agonist) for inflammation in adipose tissue [40], corticosteroids for hepatic cirrhosis [41], and *Lactobacillus* GG for alcohol-induced leaky gut, microbiota alterations, and steatohepatitis [42, 43], have been reported and examined. The available treatment options' efficacy is modest and expensive, with severe side effects. Therefore, it is sensible to explore possible preventive approaches that can suppress PPARγ in adipose tissue while also modifying the microbiota in the intestinal lumen, which should be less toxic, more potent, and affordable as functional foods.

Due to their lack of negative impacts, traditional and herbal treatments are making headway in continental therapy. The chemicals found in plants have demonstrated hypoglycemic, hypolipidemic, and anti-inflammatory benefits for NAFLD [44]. In addition, regular consumption of bioactive-rich foods may be advantageous for treating liver illnesses. Allicin is converted into diallyl sulfide (DAS), which inhibits CYP2E1 in liver disorders [45–47]. DAS lowers liver, U937 monocytic cells, astrocytic cells, and oxidative damage in rats fed with ethanol [45, 48–52]. Under acidic conditions, alliinase is inhibited, preventing the stomach's production of thiosulfate compounds such as allicin. Extract of AGE reduces glycosylated plasma albumin in a type 2 diabetes model mouse (TSOD) [53]. Allicin, diallyl disulfide, diallyl sulfide, diallyl trisulfide, S-allyl cysteine sulfoxide, S-allyl-L-cysteine, Z ajoene, and E ajoene are all components of AGE [48, 54–56]. Garlic has been used as a remedy and condiment for millennia [57]. The role of garlic as an adipose tissue inflammation inhibitor and a potential prebiotic for *Lactobacillus* (probiotic) in the presence of ethanol has not yet been investigated. However, garlic has been reported to be used as an anti-inflammatory agent for NAFLD [53, 58]. The previous article [59] on the synbiotics intervention of ethanol-induced intestinal permeability preserved the integrity and function of the colon in the presence of ethanol. To expand on our prior work on gut analysis, the present study depicted the protective effect of synbiotics in ameliorating the progression of ethanol-induced adipose tissue injury.

## Materials and methods

### Chemicals

The extracted aged garlic was delivered by Kyolic (CA, USA). *Lactobacillus rhamnosus* MTCC 1423 was purchased from The Microbial Type Culture Collection and Gene Bank (MTCC) (Chandigarh, India). Iso-butylmethylxanthine and Tri reagent was provided by Sigma‒Aldrich (MO, USA). Fetal bovine serum (FBS) was purchased from Gibco (NY, USA). The SYBR green master mix and cDNA synthesis kits were delivered by Applied Biosystems (CA, USA). Sigma supplied primer sequences manufactured commercially. Molecular biology grade ethanol with a purity of at least 99.8% and additional chemicals were procured by HiMedia Laboratories (Mumbai, India).

### Bacterial proliferation in MRS (De Man, Rogosa, and Sharpe agar) media in the presence of aged garlic extract

The effect of AGE on *Lactobacillus* growth was determined by calculating colony-forming units per milliliter and constructing a growth curve on *De Man, Rogosa, and Sharpe agar media* (MRS). Using 10^–3^, 10^–4^, and 10^–5^ dilution factors, the spread plate method was employed to calculate bacterial cell growth on MRS medium plates treated with AGE and control MRS plates without AGE supplementation. Transferring a new inoculum of 10^9^ CFU/ml into two MRS media sets constructed the growth curve. One group was supplemented with prebiotic AGE, whereas the other group received no supplementation. Until the stationary phase was reached, each set's optical density (OD) was measured at 600 nm every hour for 26 h using a UV‒visible spectrophotometer (Shimadzu, Japan).

### Culturing and maturation of the 3T3-L1 cell line

The 3T3-L1 cell line was acquired from the National Centre for Cell Sciences (NCCS) (India) and was cultured in Dulbecco's modified Eagle medium (DMEM) complete media and matured using IBMX, dexamethasone, and insulin according to the protocol mentioned in Madsen et al. [39]. These mature adipocytes were employed in subsequent ethanol and synbiotics treatments.

### Preparation of treatments for alcoholic model

#### Preparation of treatments for different experimental groups on 3T3-L1 cells

As mentioned in our previous work, the combination of AGE with *Lactobacillus rhamnosus* MTCC 1423 is used as a synbiotics [60]. In brief, an AGE working concentration of 10 μg/ml for mature 3T3-L1 adipocytes was used. In addition, cell-free supernatant was utilized for a probiotic dose on 3T3-L1, *Lactobacillus rhamnosus* MTCC 1423 (10^9^ Colony forming unit (CFU)/mL), as mentioned in our publication by Farhin et al., 2021. To generate the alcoholic model using 3T3-L1 cells, 100 mM ethanol (10 μL) for 24 h and 100 ng/ml LPS for 1 h were added before total RNA isolation [7, 60].

#### Preparation of treatments for different experimental groups of male Wistar rats

In the animal model, male Wistar rats were fed 200 mg/kg AGE, and probiotic *Lactobacillus rhamnosus* MTCC 1423 (10^9^ CFU/mL) was added in combination as a synbiotics; our publication by Farhin et al., 2021, outlined further steps of the technique for the preparation of probiotic culture [9].

### Chronic ethanol feeding of male Wistar rats and animal housing

Eight-week-old, 200–225 g male Wistar rats were provided by Zydus Pharmaceutical Industries Pvt. Ltd. (India). The Institutional Animal Ethics Committee of Ramanbhai Patel College of Pharmacy, Charotar University of Science and Technology, authorized the study protocol, including the use of animals (RPCP/IAEC/2021–22/R14). Previously, Dhara et al. [60] described the chronic ethanol feeding technique used in the investigation. To acclimate the rats, they were given free access to regular chow and water for three days following their acquisition. Before the beginning of the experiment, they were acclimatized to the liquid Liber-de Carli diet for four days.

### Development of adipocyte dysfunction induced by ethanol and evaluation of the preventative effect of probiotics and AGE treatment.

#### In vitroexperimentation using 3T3-L1 cells

**Fig. 1:**
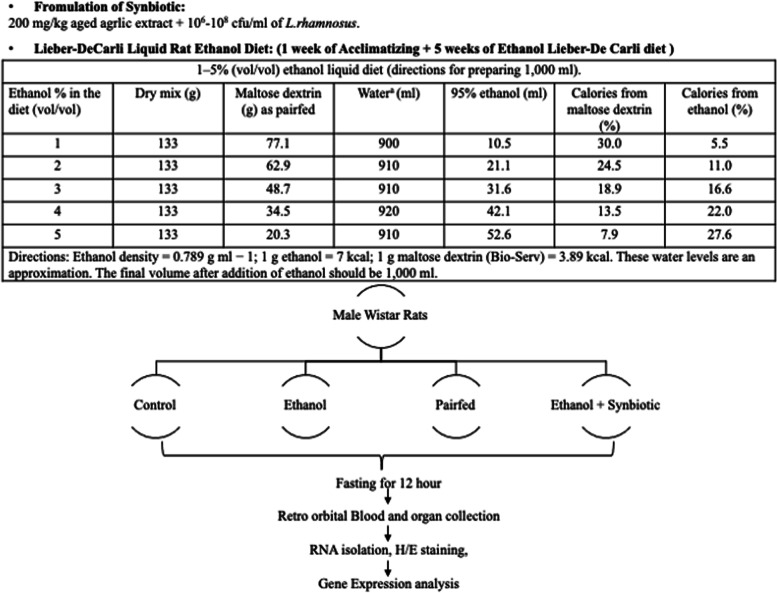
Treatment course of 3T3-L1 cells

In vitro research used four sets of mature 3T3-L1 cells. The monolayers were assessed after 1.3 × 10^5^ cells/mL were seeded in 6-well plates on Day 1. In group A, the cells received no treatment as a control; in group B, the cells were treated with LPS for one hour as a negative control; and in group C, the cells were administered 100 mM ethanol serum-free medium to induce adipocyte dysfunction. Group D: cells were exposed to 100 mM ethanol serum-free medium and LPS to induce the effect of the gut on adipocytes; Group E: Cells were exposed to serum-free media containing 10 μL/mL AGE and 10^9^ CFU/mL cell-free supernatant fraction as a synbiotics and 100 mM ethanol. Figure 1 demonstrates the experimental design.

#### In vivoexperimentation using male Wistar rats

For in vivo experiments, rats were acclimatized with Liber DeCarli for two days. Rats received full access to a 1% ethanol-Lieber DeCarli diet after acclimation. The ethanol-stimulated adipocyte disruption experiment lasted 25 days with escalating ethanol concentrations (*v/v*), as detailed in Tang. et al. 2009 [25] article. 10^9^ CFU/day *Lactobacillus rhamnosus* MTCC 1423 and 200 mg/kg AGE were delivered orally to the relevant groups. After the feeding trial of 5 weeks, fecal samples were acquired from rats in each group after 12 h of fasting. Following anesthesia with pentobarbital (Nembutal®) 40–60 mg/kg, blood was withdrawn from the vena cava of fasting rats. Adipose tissue samples were subsequently collected, fixed in RNAlater, and processed according to the method outlined by Kema et al. 2017 [48]. Serum samples were stored at -80 degrees Celsius for further analysis.

**Fig. 2:**
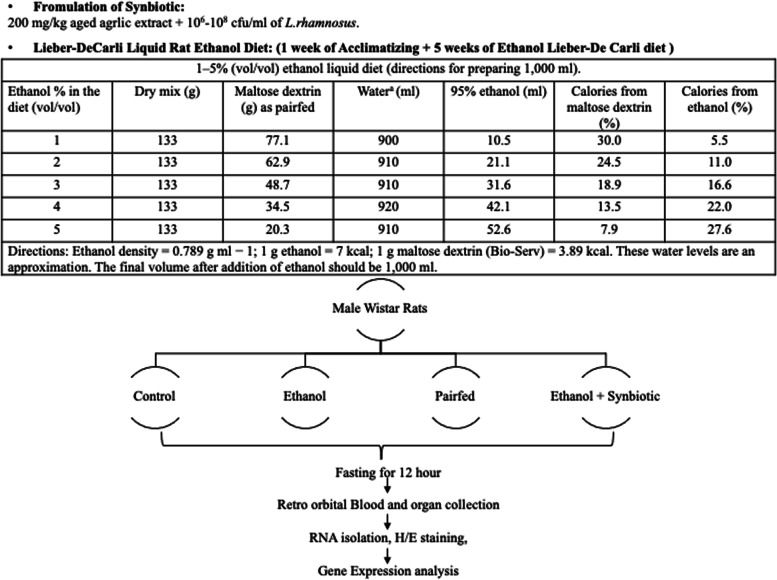
Course of treatment on 3T3-L1 cells

To evaluate the damage to adipocytes in rats (*N* = 6), they were categorized as follows: (A) the control: pellet-fed rats; (B) the ALD group: rats fed Lieber DeCarli liquid food; (C) the negative control: rats supplemented with maltodextrin in equal calories to ethanol; (D) the preventative therapy: rats fed 10^9^ CFU/day *Lactobacillus rhamnosus* MTCC 1423 and 200 mg/kg AGE as synbiotics with ethanol. The preventative therapy was administered concurrently with the Lieber DeCarli diet for 5 weeks (Fig. 2).

#### Lipid analysis of 3T3-L1 cells with oil red O staining

3T3-L1 cells were treated with 10% formalin for 1 h, and the methodology outlined by Kema et al. (2017) was then followed to determine the lipid concentration in the treatment and control groups. Finally, 1 mL of isopropanol was used to extract oil red O from the 3T3-L1 growth dish's marked cells. Absorption of the recovered stain was measured using a 510 nm UV‒Visible Spectrophotometer (Shimadzu, Japan) [61, 62].

#### Adipose tissue hematoxylin & eosin (H/E) staining

The adipose tissue for H&E staining was performed per the technique described by Kema et al. [45]. After fixation in the optimal cutting temperature (OCT) compound, the frozen adipose tissue was sliced using a Leica cryostat into 7–10 μm pieces. To identify changes in adipocyte morphology between experimental rat groups, the tissue was stained with Harris hematoxylin solution and subsequently counterstained with eosin.

#### Scanning electron microscopy

The adipose tissue sections fixed in OCT compound were carefully cut (8 ± 2 μm) and placed on a glass plate without a coverslip. Using a sputter coater (JEC-3000FC, Auto Fine Coater, Japan), a 2 nm platinum coating was applied to the smaller part of the glass slides, which were then analyzed using a scanning electron microscope (SEM; Japan Electron Optics Laboratory, JEOL 6010LA, Japan). Using a computerized image analysis system, adipocyte sizes were calculated from photomicrographs (35 ± 5 μm) produced by scanning electron microscopy of adipose tissue.

#### RNA isolation and quantitative real-time PCR

In 60 mm plates (1.5 × 10^5^ cells/mL), 3T3-L1 cells were cultured, matured, and differentiated for four weeks. A dose of 10 μL AGE, *Lactobacillus rhamnosus* MTCC 1423 (10^9^ CFU/mL), and 10 μL ethanol (100 mM) was added to the plates, and TRIzol was used to extract RNA from the cells. Formaldehyde gels were run for quality control analysis. *In *in vivo research, using 7 mg adipose tissue, RNA was extracted by the TRIzol method. The United States-made NanoDrop (Thermo Fisher Scientific) assessed RNA quantities in ng/µL and purity (A260/A280). Total RNA was treated with DNase, and cDNA was produced using a cDNA synthesis kit following the manufacturer's instructions and our previously published work [9, 52]. qRT‒PCR was then performed. Utilizing the primers listed in Tables 1 and 2, respectively. Gene expression was obtained using Agilent Mx3005P qRT‒PCR equipment (Agilent Stratagene) and SYBR/ROX Master Mix. The 18S rRNA gene served as the internal control. The results are presented as the fold-over change compared to the control group using formula 2^−ΔΔ CT.^

**Table 1:** List of Mus musculus primers for 3T3-L1 cells

Sr.No	Name of the gene	Forward Primer	Reverse primer
1	18S	ACGGAAGGGCACCACCAGGA	CACCACCACCCACGGAATCG
2	Adiponectin	GTCAGTGGATCTGACGACACCAA	ATGCCTGCCATCCAACCTG
3	Leptin	TCTCCGAGACCTCCTCCATCT	CATCCAGGCTCTCTGGCTTCT
4	Resistin	TGAGATGATTCAGTGGGTAAAGATG	TCCACCATGTAGTTTCCAGGAA
5	TNF-α,	CCCTCACACTCAGATCATCTTCT	GCTACGACGTGGGCTACAG
6	iNOS	CCCTTCCGAAGTTTCTGGCAGCAGC	GGCTGTCAGAGAGCCTCGTGGCTTTGG
7	IL6	GACAACTTTGGCATTGTGG	ATGCAGGGATGATGTTCTG
8	TLR4	ATGGCATGGCTTACACCACC	GAGGCCAATTTTGTCTCCACA
9	CYP2E1	AGCCTGAAGACTGT GATGGG	AAAGTTCCAC CGT TCTCGG
10	PPARγ	TAGGTGTGATCTTAACTGTCG	GCATGGTGTAGATGATCTCA-

**Table 2:** Rat primer list for adipose tissue

Sr No	Gene Name	Primer (Forward)	Primer (reverse)
1	18S	ACGGACCAGAGCGAAAGCAT	TGTCAATCCTGTCCGTGTCC
2	Adiponectin	AATCCTGCCCAGTCATGAAG	CATCTCCTGGGTCACCCTTA
3	Leptin	CCTGTGGCTTTGGTCCTATCTG	AGGCAAGCTGGTGAGGATCT
4	Resistin	ACTTCAGCTCCCTACTGCCA	GCTCAGTTCTCAATCAACCGTCC
5	TNF-α,	CAAGGAGGAGAAGTTCCCAA	CTCTGCTTGGTGGTTTGCTA
6	iNOS	ACAACAGGAACCTACCAGCTCA	GATGTTGTAGCGCTGTGTGTCA
7	IL6	GACTGATGTTGTTGACAGCCACTGC	TAGCCACTCCTTCTGTGACTCTAACT-
8	TLR4	GATTGCTCAGACATGGCAGTTTC	CACTCGAGGTAGGTGTTTCTGCTAA
9	CYP2E1	CTTCGGGCCAGTGTTCAC	CCCATATCTGAGTTGTGC
10	PPARγ	TAGGTGTGATCTTAACTGTCG	GCATGGTGTAGATGATCTCA-

#### Statistical analyses

All respective experiments were performed at least three times. Data from the replicates were calculated as the mean ± SD. GraphPad Prism 7 software (GraphPad Software Inc., California Corporation, San Diego, CA, USA) was used to analyze the results. The differences between all groups were analyzed using a one-way analysis of variance (ANOVA). Variations between groups were considered significant at *p*-values less than or equal to 0.05.

### Effect of synbiotics on malondialdehyde (MDA) levels in rat adipose tissue by HPLC analysis

To expand on our prior research on serum oxidative stress, 500 mg of fresh adipose tissue was homogenized at 24,000 rpm/min with 1.15% KCl according to the method described by Dhara et al. [60]. The resulting samples were analyzed using HPLC (Waters Breeze-2, USA) on an ODS2 reversed-phase column (Waters Breeze-2, USA). The mobile phase comprised 38:62 acetonitrile:0.2% acetic acid HPLC-grade water. MDA was detected at 310 nm using isocratic HPLC and a UV detector in the sample. The standard curve of 20 nmol/ml MDA solution with 1% H2SO4 was generated (TCI, Japan).

### In silico analysis to understand the effect of AGE on proteins

#### Compound-disease-target (C-D-T) network development and analysis to determine the core protein affected in the molecular mechanism of ALD

Earlier, the in vivo effect of synbiotics was evaluated on ALD-induced male Wistar rats. Alcoholic fatty liver is the most prevalent form of liver disease and can result in liver cirrhosis and cancer [63]. Therefore, the detailed mechanism of AGE against alcoholic fatty liver disease was explored by constructing compound-disease-target networks for AGE-bioactive compounds such as S-allyl cysteine sulfoxide, diallyl sulfide, diallyl disulfide, diallyl trisulfide, allicin, E-ajoene, Z-ajoene, and S-allyl-l-cysteine. The potential targets of alcoholic fatty liver were predicted using GeneCards (https://www.genecards.org/) and the DisGeNET database (http://www.disgenet.org/). GeneCards is a user-friendly and publicly available database that contains detailed information about annotated and predicted human genes. It collects data from approximately 150 online databases, including genomic, transcriptomic, proteomic, genetic, clinical, and functional data [59]. DisGeNET is a web server that comprises information about disease-associated variants and genes [64]. From the GeneCards database, alcoholic fatty liver-associated genes were identified through the “alcoholic fatty liver” keyword, and genes with scores ≥ 30 were extracted as alcoholic fatty liver-associated genes. In the case of DisGeNET, alcoholic fatty liver-associated genes were identified from disease ID C0015696.

Potential targets of AGE-bioactive components were identified using the PharmMapper (http://www.lilab-ecust.cn/pharmmapper/) tool. PharmMapper is a freely available web server developed to identify putative drug targets using a large-scale reverse pharmacophore mapping method [65]. The three-dimensional structures of selected bioactive components were obtained from the PubChem Database (https://pubchem.ncbi.nlm.nih.gov/) and submitted in sdf format to PharmMapper to acquire their putative targets. The STRING database (https://string-db.org/) version 11.0 bioinformatic tool was also used to create a protein–protein interaction (PPI) network for AGE-bioactive components and alcoholic fatty liver with a confidence score of 0.4. [66]. Using Cytoscape 3.8.0 software, these networks were utilized to create C-D-T networks for each bioactive component of AGE against alcoholic fatty liver [66].

#### Molecular docking of PPARγ protein as it is most affected in the molecular mechanism of ALD

The 3D structure of the PPARγ protein (PDB ID 7AWC) was found in the Protein Data Bank (PDB ID 7AWC). The protein was also crystallized in the presence of a rosiglitazone inhibitor. Before the docking technique, the coordinates of the rosiglitazone binding site on PPARγ were identified and deleted. The crystalline protein structure was modified by adding hydrogen atoms and Kollman charges. The.pdbqt format was used to save the processed protein structure. PubChem was used to find all of the ligands. The torsion tree and the number of rotatable bonds were defined to optimize the ligand molecules. The ligand data were also stored in pdbqt format for additional docking studies. Grid creation is crucial in molecular docking simulation because it guides ligands to the protein and #39 active site. The grid spacing was set at 0.375 inches (default). The values for the center grid boxes were set to 41.865, 3.643, and 83.672, respectively. In the x, y, and z dimensions, the number of grid points was set to 40 40 40. In the current study, all ligands were docked with the PPARγ protein in the binding location of the rosiglitazone inhibitor. AutoDock 4.2.6 software was used to dock the ligands with PPARγ [67]. The ligand interactions with the PPARγ protein were visualized and examined using the BIOVIA Discover Studio program.

## Results

### The presence of prebiotic AGEs enhances the growth of Lactobacillus rhamnosus MTCC 1423

**Fig. 3:**
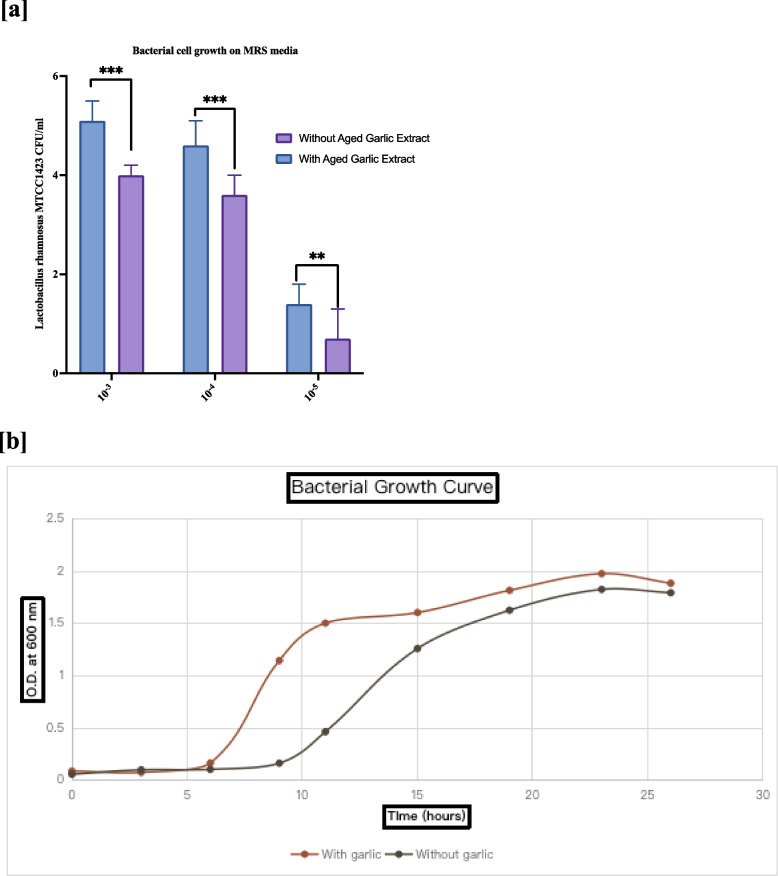
The presence of AGE enhances the growth of *Lactobacillus rhamnosus* MTCC 1423 **a** *Spread Plate Method for Bacterial colonies:* The spread plate method for bacterial growth in the dilution factor 10^–3, 4, and 5^ group with prebiotic AGE is increased compared to the absence of AGE. Significance (*p*-value) is determined as * *p* < 0.05, ** *p* < 0.01, *** *p* < 0.001, and **** *p* < 0.0001 in comparison between with aged garlic extract and without aged garlic extract. **b ***Growth Curve:* Bacterial Growth curve of *Lactobacillus rhamnosus* MTCC 1423 in MRS broth

The effect of AGE on the growth of the *Lactobacillus* probiotic bacterium was elucidated using the growth curve method. The spread plate technique for determining bacterial growth on MRS media is more sensitive at dilution factors more significant than 10^–3.^ The development of *Lactobacillus rhamnosus* MTCC 1423 increased in the presence of the prebiotic AGE compared to that in the absence of AGE, as shown in Fig. 3a. The bacterial growth curve to determine the log phase of *Lactobacillus rhamnosus* MTCC 1423 demonstrated in Fig. 3b that an early log phase was achieved at approximately 6 h in the presence of AGE compared with AGE absence. This signifies that the combination of AGE and *Lactobacillus rhamnosus* MTCC1423 is efficient as a synbiotics.

### Synbiotics enhance lipid accumulation in anin vitroALD model using 3T3-L1 cells

**Fig. 4:**
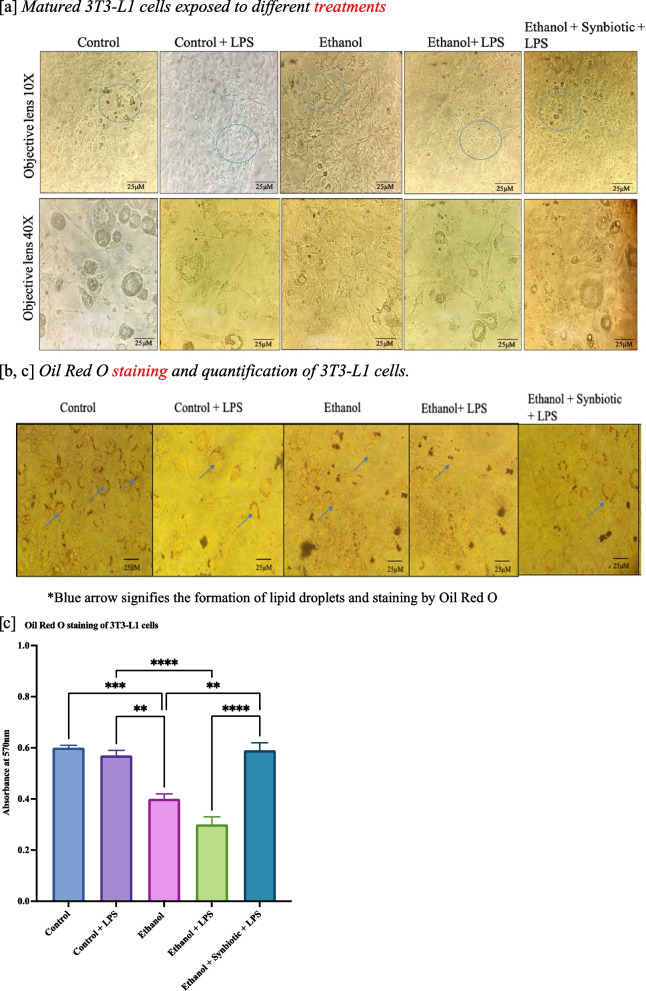
Synbiotics improve adipocyte morphology, lipogenesis, and gene expression in 3T3-L1 cells. Matured 3T3-L1 ethanol- and synbiotics-exposed cells exhibited elevated lipogenesis. All images were taken at 10X and 40X. [b] Oil Red O staining of 3T3-L1 treated cells with A control, B Control + LPS, C Ethanol, D Ethanol + LPS, I Ethanol + synbiotics + LPS for 24 h. [c] Spectrophotometric study of lipogenesis in 3T3-L1 adipocytes using the oil red O method with IP as a blank. Statistical significance was analyzed as described in Statistical analyses section

The organ responsible for the storage of lipids is adipose tissue. As shown in Fig. 4a, exposure of differentiated 3T3-L1 cells to ethanol dramatically reduced lipogenesis (lipid droplets). As determined by the Oil red O stain extraction method, however, treating these ethanol-exposed cells with the synbiotics combination increased lipogenesis within the cells (Fig. 4b and c). Oil red extracted with isopropanol (IP) produced an absorbance of 0.06 ± 0.004 for control cells stained with oxygen. The absorbance value of oil red O for ethanol-exposed 3T3-L1 cells was 0.4 ± 0.002; ethanol-exposed synbiotics-treated cells had an absorbance value of 0.6 ± 0.013. The absorbance values of ethanol and ethanol-exposed symbiotically treated samples differed significantly. This may be due to the synbiotics capacity to stimulate adipocyte cell division. These results indicate that the synbiotics combination effectively rectifies the adipocyte morphology and lipogenesis defects caused by ethanol.

### Synbiotics regulate the inflammatory response upon ethanol exposure in 3T3-L1 cells

**Fig. 5:**
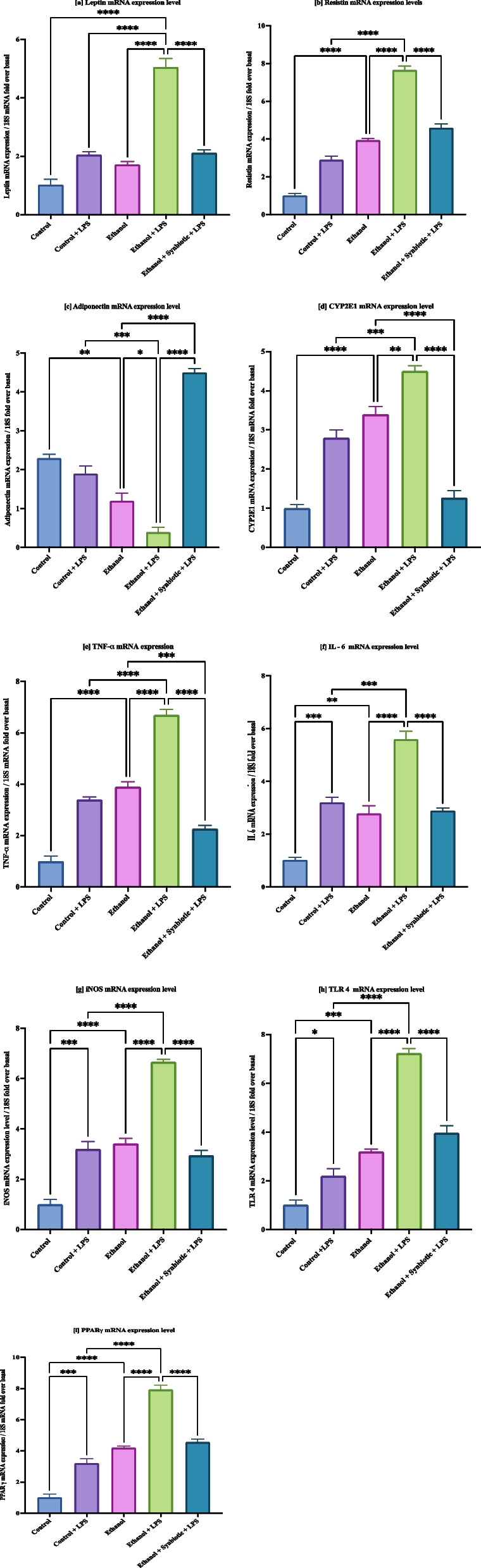
Synbiotics regulate the inflammatory response upon ethanol exposure in 3T3-L1 cells. qRT‒PCR gene investigation of [**a**] leptin, [**b**] resistin, [**c**] adiponectin, [**d**] CYP2E1, [**e**] TNF-α [**f**] IL-6, [**g**] iNOS, [**h**] TLR4, and [**i**] PPARγ from ethanol- and synbiotics-treated 3T3-L1 cells. qRT‒PCR was performed in triplicate. After the normalization of 18S in each sample, the gene expression level was determined as per the statistical analysis described in Statistical analyses section

Compared to the control cells, ethanol-exposed 3T3-L1 cells significantly increased CYP2E1 mRNA expression. However, treating the ethanol-exposed cells with the synbiotics reduced the mRNA expression levels of CYP2E1 (Fig. 5d). Inflammatory adipokine mRNA expression levels, such as leptin, resistin, and inflammatory markers, such as TNF-α, inducible nitric oxide synthase (iNOS), IL6, and PPARγ, were upregulated upon ethanol exposure in 3T3-L1 cells compared with the control group. This illustrates that the synbiotics treatment decreases the gene expression of inflammatory adipokines and cytokines in ethanol-exposed 3T3-L1 adipocyte cells in contrast to ethanol plus AGE and ethanol-treated 3T3-L1 adipocyte cells. In ALD, a leaky gut triggers the TLR4 pathway, which is downregulated by synbiotics. The levels of anti-inflammatory adipokines and adiponectin decrease in ethanol-exposed cells. Upon administering the synbiotics and AGE doses to the ethanol-exposed 3T3-L1 cells, the level of adiponectin increased, suggesting a decrease in the inflammation resulting from exposure to ethanol in 3T3-L1 cells (Fig. 5 a-c,e,f-i).

### Synbiotics Effect on ethanol-induced reduction in adipocyte size, adipose tissue mass, and lipid accumulation in male Wistar Rats

**Fig. 6:**
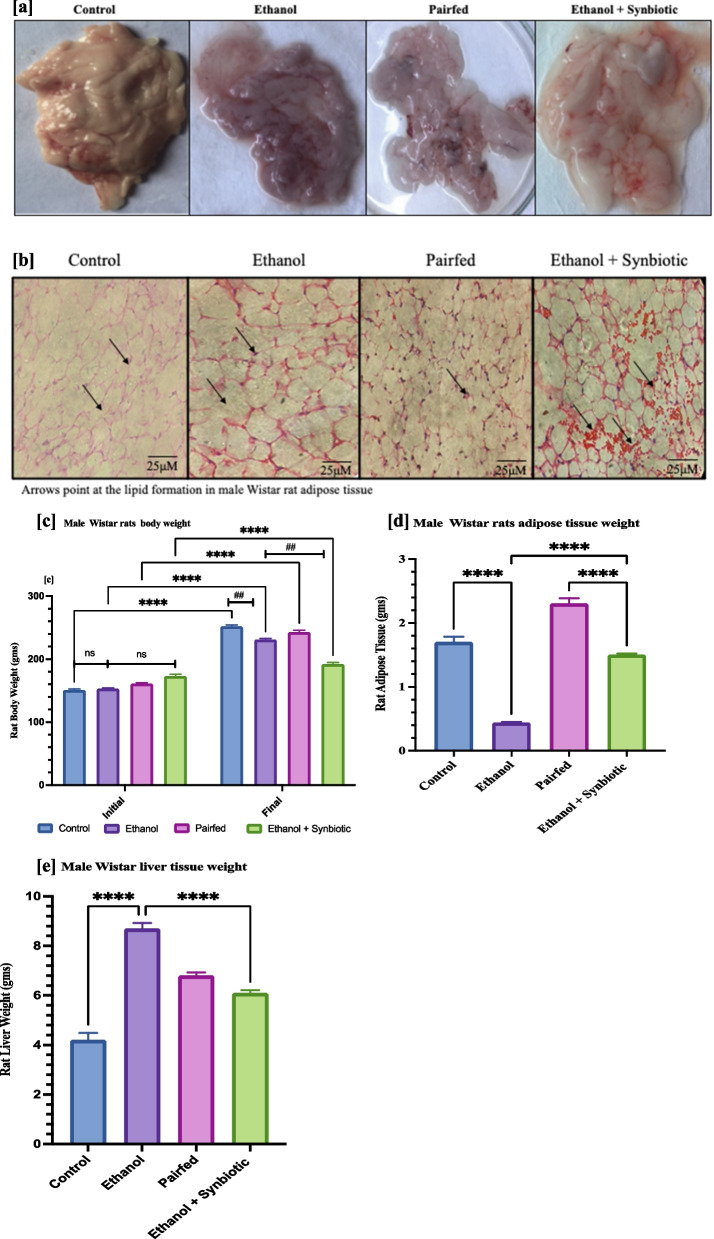
In male Wistar rats, the effect of a synbiotic on ethanol-induced adipocyte size, adipose tissue mass, and lipid accumulation. **c** Graph representing the rat body weight in the ALD model. * Signifies the difference between the initial and final body weight of the male Wistar rat, while # signifies the difference with reference to ethanol and synbiotic group in the final weight. Non-significance within the initial groups is denoted by ns [**d**] Graph represents the male Wistar rat adipose tissue weight in the ALD model. **e** Graph represents the significance of the male Wistar rat liver weight in the ALD model. Significance (*p*-value) was determined by Tukey’s multiple comparison tests as * *p* < 0.05, ^** or ##^*p* < 0.01, *** *p* < 0.001, and **** *p* < 0.0001 in the comparison ethanol group

Ethanol treatment of male Wistar rats decreased the adipose tissue mass (Fig. 6). However, feeding the rats with synbiotics added to the ethanol feeding diet (200 mg/kg body weight plus 10^8^ CFU/ml *Lactobacillus rhamnosus* MTCC 1423) increased the adipose tissue mass. H/E sections of adipose tissue in ethanol-fed rats revealed some modification of adipocyte morphology. The cells presented a distorted phenotype, and the shape of the cells was deformed. The tissue section of rats fed a synbiotics diet during the ethanol feeding protocol portrayed well-structured adipocytes with increased lipid droplets compared to the AGE and ethanol groups (Fig. 9). The staining results indicated that synbiotics treatment augments adipocyte morphology and adipose tissue mass compared to ethanol treatment.

### Scanning electron microscopy (SEM) images reveal adipose tissue morphology

**Fig. 7:**
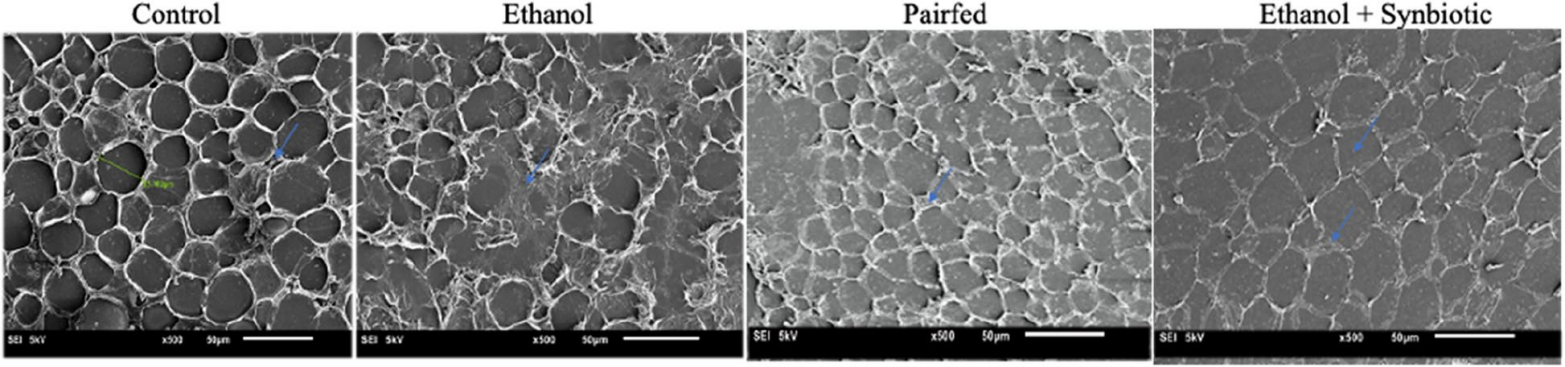
Scanning electron microscope images illustrating the morphology of adipose tissue. Visceral adipose tissue (VAT) of Wistar male rats for the control, ethanol, pairfed, and ethanol + synbiotics groups

The scanning electron images depict (or indicate) that the adipocyte morphology is intact in the control VAT group. In contrast, upon exposure to ethanol, the adipocyte cell wall is disrupted, leading to the apoptosis of adipocytes. However, cells at 50 μm with ethanol plus synbiotics revealed that the cells were intact, but the cell size was more significant than the VAT ethanol group. Thus, the SEM images signify that the morphology of the adipose tissue in Wistar rats was restored by synbiotics administration, as shown in Fig. 7.

### Synbiotics ingestion in Wistar rats regulates ethanol-induced modifications in the inflammatory response of adipose tissue

**Fig. 8:**
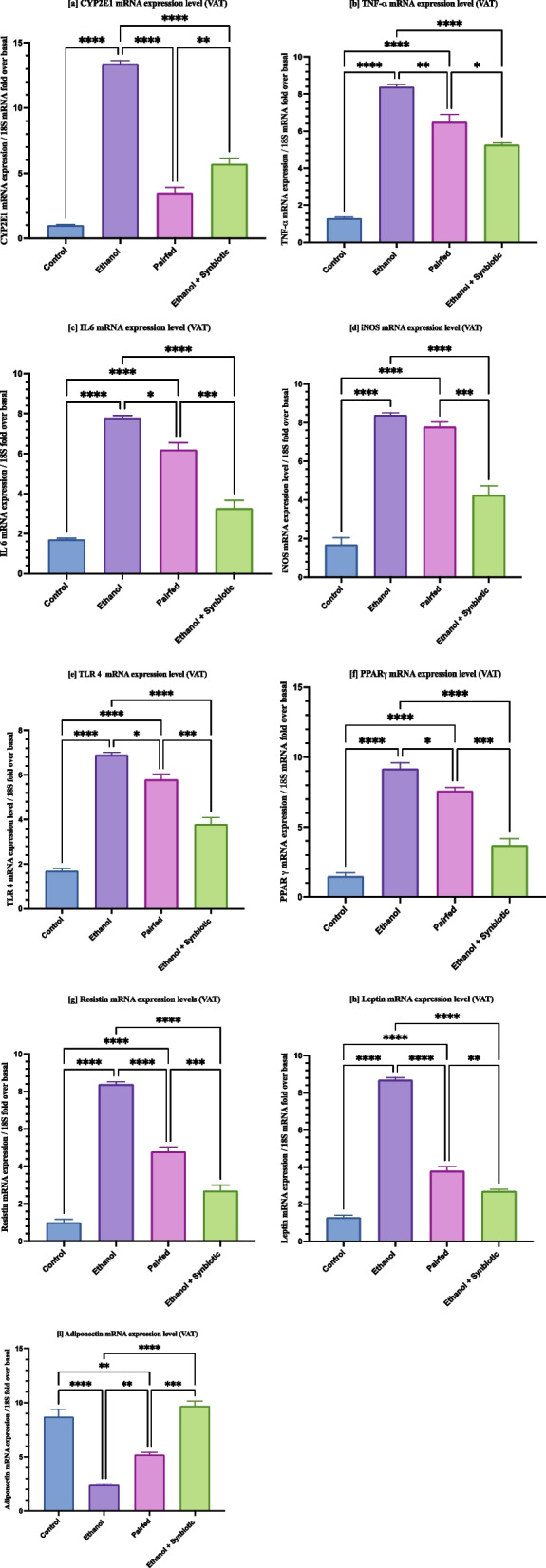
qRT‒PCR gene data reveal that synbiotics ingestion by male Wistar rats affects ethanol-mediated inhibition of the inflammatory pathway in adipose tissue represented as [**a**] CYP2E1 [**b**] TNF-α [**c**] IL6 [**d**] iNOS [**e**] TLR4 [**f**] PPARγ [**g**] resistin [**h**] leptin [**i**] adiponectin. qRT‒PCR was performed in triplicate. Each sample's normalizing 18S was taken into consideration. As indicated in Statistical analyses section, the gene expression level was calculated and given as relative mRNA expression

Alcohol consumption disrupts the release of adipokines from adipose tissue, resulting in dysregulation of adipose tissue metabolism and alteration of phenotype [22, 68–70]. The alcoholic model of rodents demonstrated an upregulation of pro-inflammatory cytokines such as TNF-α, IL6, leptin, and resistin and the downregulation of anti-inflammatory cytokines such as adiponectin [24, 71, 72]. Our results reveal that feeding male Wistar rats a synbiotics reduced the levels of pro-inflammatory genes such as TNF-α, leptin, resistin, and iNOS in adipose tissue of the leaky rat gut because chronic alcohol consumption leads to the diffusion of LPS into portal vein circulation. These factors further trigger the TLR4 signaling pathway, resulting in the upregulation of the production of inflammatory cytokines [73–75]. Treatment with synbiotics reduces the levels of TLR4 expression in the VAT of rats. The CYP2E1 enzyme involved in ethanol metabolism considerably decreased in ethanol-exposed synbiotics-fed rats compared to ethanol-fed rats. The upregulation of anti-inflammatory adiponectin was observed in the adipose tissue of rats fed with ethanol-exposed synbiotics group compared to only ethanol-fed rats (Fig. 8). Moreover, these results suggest that the synbiotics combination effectively regulates ethanol-induced inflammation.

### High-performance liquid chromatography (HPLC) for MDA analysis of rat adipose tissue

**Fig. 9:**
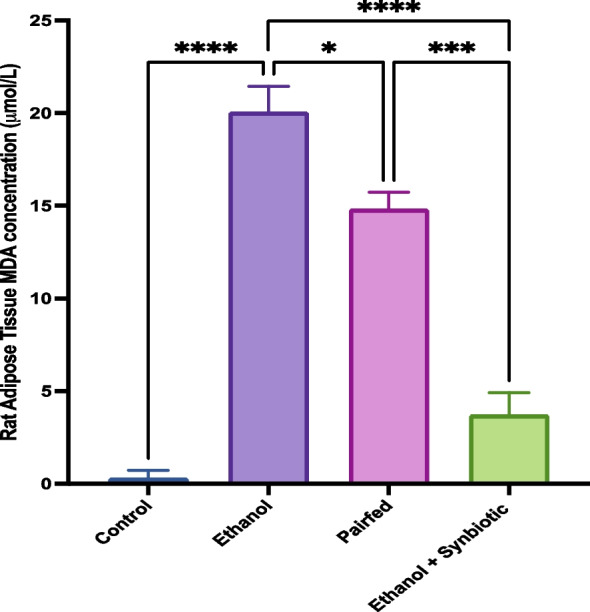
On 3T3-L1 cells, a graphical representation of the synbiotics effect on MDA concentration by the HPLC method. The statistical analysis was calculated as mentioned in Statistical analyses section

Supporting the published rat serum MDA estimation in Dhara et al. [60]. MDA levels in the adipose tissue of male Wistar rats treated with ethanol were 19.69 ± 1.75 µM/L, compared to 0.60 ± 0.4 µM/L in the control group. Figure 9 shows that adipose tissue with the synbiotics and ethanol had significantly lower MDA levels of 2.21 ± 1.98 µM/L than the ethanol and pairfed (13.31 ± 2.3 µM/L) groups.

### To understand the effect of AGE on ALD-associated proteins using Network Pharmacology approach

#### Compound-disease-target (C-D-T) networks of AGEs against alcoholic fatty liver

**Fig. 10:**
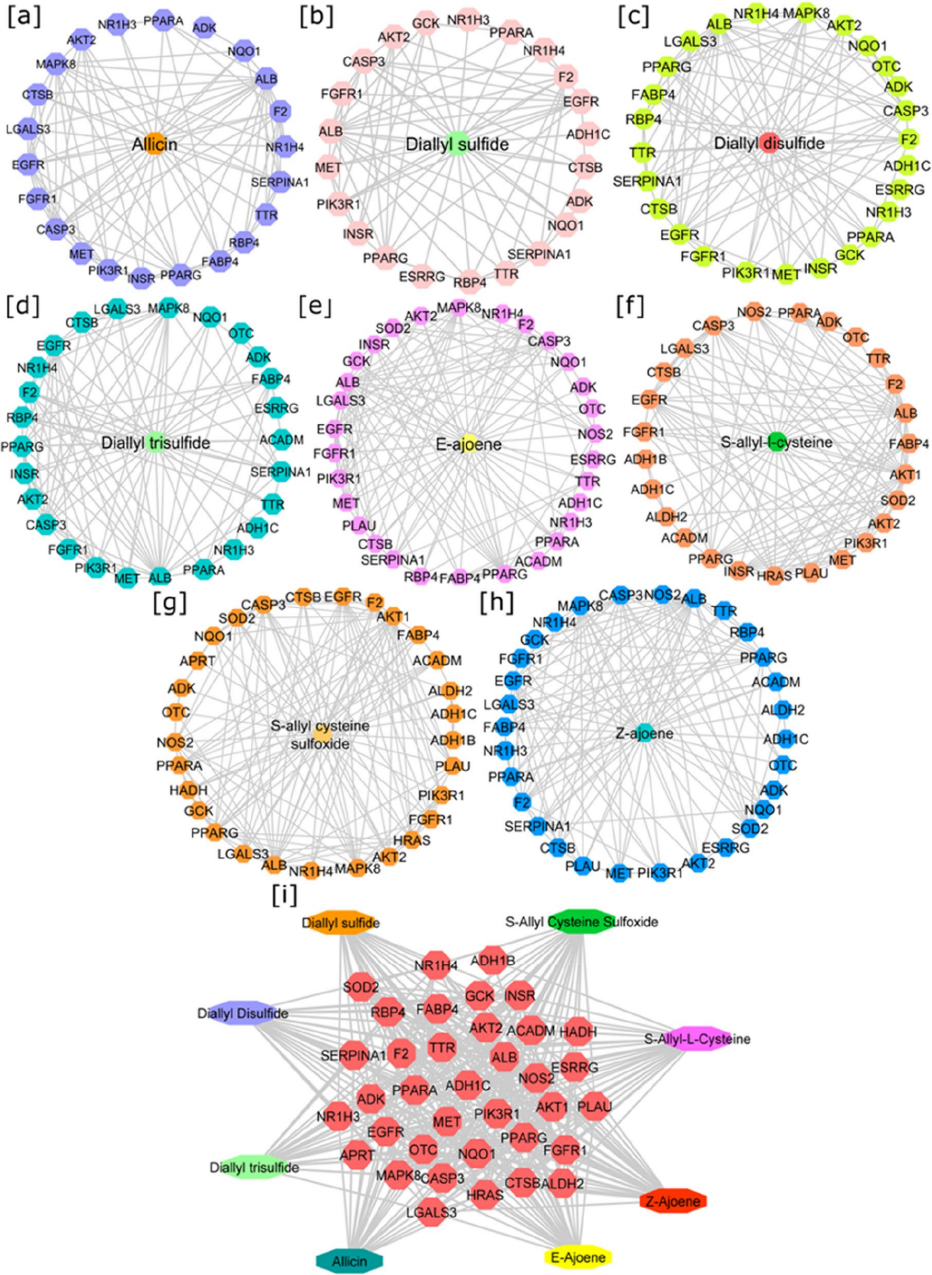
Compound-disease-target (C-D-T) networks of AGE-bioactive constituents: [**a**] Allicin, [**b**] Diallyl sulfide, [**c**] Diallyl disulfide, [**d**] Diallyl trisulfide, [**e**] E-ajoene, [**f**] S-allyl-L-cysteine, [**g**] S-allyl cysteine sulfoxide, [**h**] Z-ajoene and [**I**] combined network for 36 target proteins

Network pharmacology is an emerging area for drug target prediction to determine the drug-gene-disease interaction mechanism [76]. A total of 188, 230, 251, 219, 299, 299, 299, and 299 potential gene targets were obtained from PharmMapper for AGE bioactive compounds: allicin, diallyl disulfide, diallyl sulfide, diallyl trisulfide, S-allyl cysteine sulfoxide, S-allyl-L- cysteine, Z ajoene, and E ajoene, respectively. After eliminating duplicates, 208 potential gene targets for alcoholic fatty liver were identified from the DisGeNet and Gene Cards databases. Using the STRING database, these identified potential bioactive compounds and alcoholic fatty liver targets were used to construct a C-D-T network for each compound in Cytoscape software. The resultant C-D-T networks had 23 nodes/76 edges, 27 nodes/104 edges, 27 nodes/103 edges, 23 nodes/94 edges, 31 nodes/132 edges, 27 nodes/125 edges, 31 nodes/128 edges, and 30 nodes/145 edges against alcoholic fatty liver for allicin, diallyl disulfide, diallyl sulfide, diallyl trisulfide, S-allyl cysteine sulfoxide, S-allyl-L-cysteine, Z ajoene, and E ajoene, respectively. These results indicated that allicin, diallyl disulfide, diallyl sulfide, diallyl trisulfide, S-allyl cysteine sulfoxide, S-allyl-L-cysteine, Z ajoene, and E ajoene could target 22, 26, 26, 22, 30, 26, 30, and 29 proteins of alcoholic fatty liver, respectively, by excluding the compound node. Additionally, a combined C-D-T network was constructed to visualize all the AGE bioactive compounds targeting 36 alcoholic fatty liver-associated proteins. However, targeting the core targets may disrupt the whole C-D-T network. Hence, the node degree topological parameter of combined C-D-T was analyzed using the network analyzer tool of Cytoscape. Node degree analysis revealed that PPARγ possessed the highest node degree in the combined C-D-T network, as shown in Fig. 10. Therefore, the PPARγ core target protein was selected for the molecular docking procedure.

#### AGE showed affinity as an inhibitor for PPARγ as a core target protein for ALD prevention

**Fig. 11:**
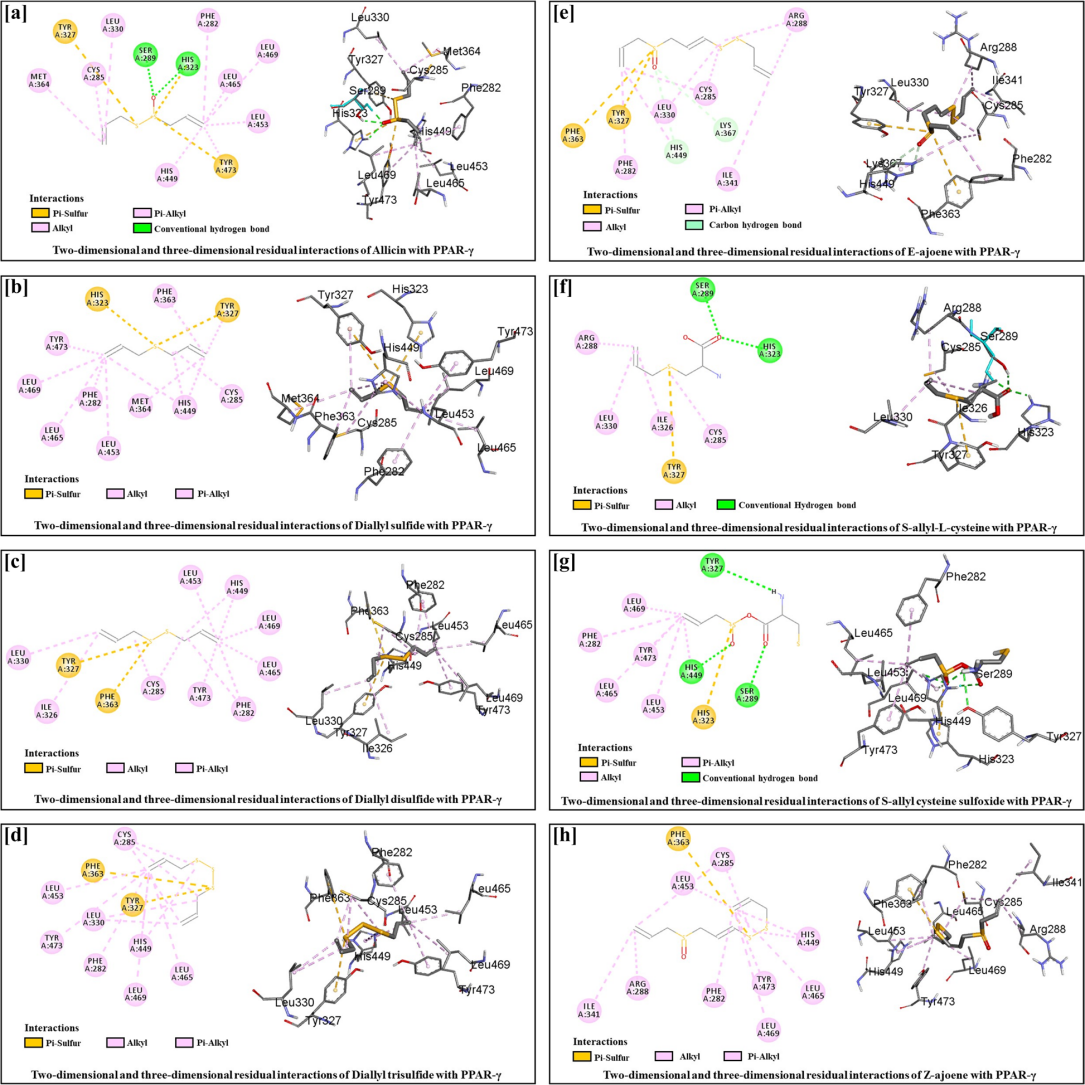
Molecular docking of AGE-bioactive components with PPARγ: Two-dimensional and three-dimensional residual interactions of [**a**] Allicin, [**b**] Diallyl sulfide, [**c**] Diallyl disulfide, [**d**] Diallyl trisulfide, [**e**] E-ajoene, [**f**] S-allyl-L-cysteine, [**g**] S-allyl cysteine sulfoxide and [**h**] Z-ajoene with PPARγ

Ethanol initiates inflammatory pathways by producing proinflammatory cytokines, further producing oxidative stress. The suppression of PPARγ inhibits various inflammatory responses, which can function as protective mechanisms that delay the progression of ALD. Molecular docking is a computational method commonly used for predicting ligands' experimental binding mode and affinity at the binding pocket of the target protein [77]. Molecular docking was employed to analyze the interactions and binding energy of bioactive components of AGEs with the core target protein (PPARγ) of the combined C-D-T network. The binding affinities of S-allyl cysteine sulfoxide, diallyl sulfide, diallyl disulfide, diallyl trisulfide, allicin, e-ajoene, z-ajoene, and s-allyl-l-cysteine with PPARγ were -5.4, -3.8, -3.9, -4.0, -4.6, -4.9, -4.9, and -4.9 kcal/mol, respectively. S-allyl cysteine sulfoxide showed higher binding affinity to bind and downregulate PPARγ protein when compared to other components. The hydrogen and hydrophobic interaction profiles of allicin, diallyl disulfide, diallyl sulfide, diallyl trisulfide, S-allyl cysteine sulfoxide, S-allyl-L-cysteine, Z ajoene and E ajoene with the receptor PPARγ and their distances are depicted in Fig. 10. The AGE-bioactive components showed a different but negative binding affinity for PPARγ, indicating that these components can target the PPARγ core protein. Therefore, the effect of AGE on the expression of PPARγ protein was further validated by experimental analysis, as shown in Fig. 11.

## Discussion

ALD inflammation affects multiple organs, including the gut and adipose tissue. Thus, finding a combinational molecule with greater efficacy to target both gut and adipose tissue is essential. According to a recent review of cirrhosis, liver damage is related to altered gut microbiota and intestinal epithelial function [13]. As per our recent work, the synbiotics preventive approach of AGE and *Lactobacillus rhamnosus* MTCC 1423 protects the gut lumen effectively by preventing ALD progression [60].

Alcohol also affects a patient's weight. The role of adipose tissue was evaluated to understand the molecular mechanism of synbiotics in ALD. A significant organ plays a dual role as a metabolic and endocrine organ to regulate energy and lipogenesis. [78–80]. Chronic drinking of alcohol harms adipose tissue metabolism, leading to steatosis progression to steatohepatitis. Accumulation of visceral fat and inflammation of adipose tissue are positively correlated with liver damage in alcoholic patients [20, 21, 26, 81]. Chronic alcohol consumption causes adipocyte death and decreases anti-inflammatory adiponectin levels [68, 69, 82, 83]. Lipolysis of adipose tissue results in the loss of white adipose tissue and a rise in circulating free fatty acids upon chronic ethanol use [26, 82]. This study assessed synbiotics intervention in the adipose tissue of in vitro and in vivo models of ALD.

Chronic alcohol consumption results in the inflammatory process and oxidative stress followed by C1q/Bid pathway activation by CYP2E1, causing apoptosis of adipocytes that results in the inflammation of the rat adipose tissue, further leading to loss of function and dysregulation of metabolism of adipose tissue [22]. Mature 3T3-L1 adipocytes overexpress the CYP2E1 enzyme, producing enhanced oxidative stress when exposed to ethanol-containing media [23, 83]. Preventive regulation of cytokine systems offers the potential for substantial alterations in adipose tissue function. Adipose tissue derives its cytokines from adipocytes, preadipocytes, and other cell types. According to mRNA expression studies, adipocytes can produce TNF-α, IL-1b, and IL-6. Adipocyte cytokine secretion appears comparable to that of other cell types. In obese individuals, IL-6 concentrations are moderately higher, according to the consensus [84].

Recent research has demonstrated that LPS injection boosts iNOS activity and protein levels in the epididymal adipose tissue of rats. This suggests that adipose tissue is a possible source of NO generation during endotoxemia. Nonetheless, the precise involvement of adipose tissue in circulatory NO generation in endotoxic shock is still unknown. In addition, the method by which iNOS is induced in adipose cells needs to be better understood [85]. In this study, the mRNA expression of CYP2E1, TNF-α, IL-6, and iNOS was significantly suppressed by the synbiotics compared to the LPS, ethanol + LPS, and ethanol groups in 3T3-L1 cells. In support of the in vitro findings, the mRNA expression of CYP2E1, TNF-α, iNOS, and IL-6 in the adipose tissue of Wistar male rats was significantly reduced in the synbiotics group compared to the Pairfed (negative control) and ethanol groups.

Regarding the inflammatory response of adipose tissue, TLR4 has attracted the most significant interest. Fetuin-A has been associated with the interaction between TLR4 and fatty acids. Adipose tissue produces a substantial amount of Fetuin-A, identified as an endogenous ligand of TLR4 [86]. This is similar to the transition that develops in adipose tissue during obesity. Inactivation of TLR4 decreases adipose tissue inflammation, but its effects on whole-body insulin levels are inconsistent [87]. Correspondingly*, *in vitro and in vivo results in decreased TLR 4 mRNA expression upon synbiotics administration in ALD models compared to ethanol.

Moreover, adipokines such as resistin, visfatin, leptin, and chemerin levels increase in a dose-dependent manner. A decrease in adiponectin concentrations was detected in ethanol-fed male Wistar rat VAT [71]. Likewise, in this study, the mRNA expression of inflammatory markers such as resistin and leptin in the differentiated 3T3-L1 and adipose tissue of male Wistar rats was increased when exposed to ethanol and reduced by synbiotics administration even in the presence of ethanol and LPS. Our results are consistent with earlier studies on ethanol consumption [25, 88–90].

In contrast, obesity and its accompanying diseases reduce the secretion of adiponectin, an adipokine with insulin-sensitizing and anti-inflammatory properties. Recent data suggest that adiponectin protects against vascular dysfunction caused by obesity and diabetes via its many positive effects on glucose, lipid metabolism, and vascular function [91]. The current work determined that synbiotics therapy efficiently lowers inflammatory markers and elevates the anti-inflammatory adipokine and adiponectin in differentiated 3T3-L1 cells and adipose tissue of Wistar male rats. PPAR γ is abundantly expressed in adipose tissue and is integral to adipose tissue function. PPAR γ activation is related to potentially favorable effects on the expression and secretion of adiponectin, resistin, leptin, IL-6, TNF-α, PAI-1, MCP-1, and angiotensinogen [92]. Synbiotics administration significantly reduces PPARγ in ethanol-exposed mature 3T3-L1 cells and adipose tissue compared to the pairfed and LPS groups.

Multiple factors influence ALD incidence and progression. ALD is characterized by the peroxidation of cellular lipids, which generates 4-hydroxynonenal (4-HNE) and malondialdehyde (MDA) due to oxidative stress. The level of lipid peroxidation in adipose tissue, as determined by malondialdehyde (MDA) using the HPLC method, decreased significantly in the synbiotics treatment group compared to the ethanol and pairfed groups of male Wistar rats, which correlates with our continued research on the colon in ALD.

In support of the inflammatory panel study, morphological changes indicate lipolysis in differentiated 3T3-L1 cells subjected to ethanol and LPS. Oil red O indicated a reduction in lipid droplets. Synbiotics treatment demonstrated that the adipocyte cell membrane and lipid droplets were remarkably preserved. Similar results were acquired from the Liber-Decarli ethanol-induced model of male Wistar rats; the ethanol-treated group's adipose tissue mass was severely reduced. However, the synbiotics treatment considerably maintained the adipose tissue mass. H/E staining and SEM images demonstrated that synbiotics treatment also preserved the cell membrane of adipocytes effectively compared to ethanol-fed rats. These results indicate that synbiotics have the potential to be preventive agents in halting the progression of ALD.

No FDA-approved drug is available to treat ALD due to the complexity of dosage, compatibility, and disease progression in the long run. Therefore, network pharmacology is vital in understanding potential preventive prospects in disease conditions with low research costs and cycles. In previously reported studies, the effects of AGEs against fatty liver disease have been investigated by in vivo methods [58, 93–96]. In the present study, synbiotics maintained adipose tissue inflammation, and an in silico approach was employed initially to evaluate the pharmacokinetic properties and explore the pharmacological mechanism of AGE against alcoholic fatty liver. Additionally, Ried et al. [95] examined whether supplementation with kyolic AGE was highly tolerated and showed a high safety profile with multiple cardiovascular benefits in treating hypertensive patients.

Furthermore, C-D-T networks were constructed to identify the preventive targets of bioactive components of AGEs against alcoholic fatty liver. However, the combined C-D-T network analysis revealed that the bioactive components of AGEs could target 36 alcoholic fatty liver-associated proteins. Moreover, AGE is beneficial for preventing alcoholic fatty liver, as it targets multiple proteins. According to the topological node degree analysis, PPARγ is a core target protein of the combined C-D-T network. Therefore, the binding efficiency of the bioactive components of AGEs with PPARγ was validated by molecular docking simulation. Molecular docking results revealed that the binding energy of each bioactive component of AGE for PPARγ was negative, which indicates that AGE downregulates the expression of PPARγ. The downregulation of the PPARγ gene in ALD was further correlated by mRNA expression analysis.

This is the first study to investigate the effect of probiotics in combination with garlic, a potentially novel synbiotics therapeutic agent for downregulating PPARγ expression in lipid metabolism in an ethanol-induced ALD model. In addition, this study only examined the early phases of ALD. The effect of synbiotics on the chronic stages, i.e., longer than 5 weeks, of ALD has yet to be assessed.

## Conclusion

Chronic alcohol consumption modifies adipose tissue metabolism, including inappropriate stimulation of lipolysis, lipid peroxidation, adipokine production, and adipokine expression, promoting an inflammatory milieu. We report that synbiotics downregulate the PPARγ gene and upregulate adiponectin levels, thereby inhibiting the release of proinflammatory cytokines such as resistin, leptin, CYP2E1, TNF-α, iNOS, and IL6 to prevent adipose tissue inflammation and the progression of ALD. Future possibilities include analyzing the effect of synbiotics on the protein markers that play a crucial role in the onset of ALD.

## Data Availability

The authors confirm that the data supporting the findings of this study are available within the manuscript.
